# Possible type 1 diabetes risk prediction: Using ultrasound imaging to assess pancreas inflammation in the inducible autoimmune diabetes BBDR model

**DOI:** 10.1371/journal.pone.0178641

**Published:** 2017-06-12

**Authors:** Frederick R. Roberts, Clinton Hupple, Elaine Norowski, Nicole C. Walsh, Natalia Przewozniak, Ken-Edwin Aryee, Filia M. Van Dessel, Agata Jurczyk, David M. Harlan, Dale L. Greiner, Rita Bortell, Chaoxing Yang

**Affiliations:** 1 FuJiFilm VisualSonics, Toronto, Ontario, Canada; 2 Program in Molecular Medicine, University of Massachusetts Medical School, Worcester, Massachusetts, United States of America; 3 Department of Medicine, University of Massachusetts Medical School, Massachusetts, United States of America; La Jolla Institute for Allergy and Immunology, UNITED STATES

## Abstract

**Background/Aims:**

Studies of human cadaveric pancreas specimens indicate that pancreas inflammation plays an important role in type 1 diabetes pathogenesis. Due to the inaccessibility of pancreas in living patients, imaging technology to visualize pancreas inflammation is much in need. In this study, we investigated the feasibility of utilizing ultrasound imaging to assess pancreas inflammation longitudinally in living rats during the progression leading to type 1 diabetes onset.

**Methods:**

The virus-inducible BBDR type 1 diabetes rat model was used to systematically investigate pancreas changes that occur prior to and during development of autoimmunity. The nearly 100% diabetes incidence upon virus induction and the highly consistent time course of this rat model make longitudinal imaging examination possible. A combination of histology, immunoblotting, flow cytometry, and ultrasound imaging technology was used to identify stage-specific pancreas changes.

**Results:**

Our histology data indicated that exocrine pancreas tissue of the diabetes-induced rats underwent dramatic changes, including blood vessel dilation and increased CD8+ cell infiltration, at a very early stage of disease initiation. Ultrasound imaging data revealed significant acute and persistent pancreas inflammation in the diabetes-induced rats. The pancreas micro-vasculature was significantly dilated one day after diabetes induction, and large blood vessel (superior mesenteric artery in this study) dilation and inflammation occurred several days later, but still prior to any observable autoimmune cell infiltration of the pancreatic islets.

**Conclusions:**

Our data demonstrate that ultrasound imaging technology can detect pancreas inflammation in living rats during the development of type 1 diabetes. Due to ultrasound’s established use as a non-invasive diagnostic tool, it may prove useful in a clinical setting for type 1 diabetes risk prediction prior to autoimmunity and to assess the effectiveness of potential therapeutics.

## Introduction

Type 1 diabetes is caused by the autoimmune destruction of insulin-producing β-cells within the pancreatic endocrine islets, and both genetics and the environment play etiological roles [1–3]. A mechanistic understanding of the causes of type 1 diabetes remains elusive due to inaccessibility of the target organ—pancreas. Biopsies of the pancreata of type 1 diabetic patients have been performed, but these studies have been halted due to severe adverse side effects [4, 5]. Thanks to the increasing availability of cadaveric pancreas specimens from organizations, such as the Network of Pancreatic Organ Donors (nPOD), many unexpected type 1 diabetes pathologic features in the human pancreas have recently been revealed [6–8].

In general, type 1 diabetes is caused by autoimmune destruction of β-cells within the pancreatic islets. Therefore, because the islets constituent only a small fraction (~2%) of the entire pancreas, it was at first surprising that pancreata from type 1 diabetic organ donors showed significant reductions in pancreas weight and volume compared to those of non-diabetics [9]. Moreover, immunostaining of human pancreatic tissue showed many type 1 diabetic donors with higher CD8 T cell density in the *exocrine* tissue [10], which is not typically an autoimmune target, although circulating autoantibodies against exocrine proteins and enzymes have been reported [11]. Conversely, the expected immune cell infiltration in pancreatic islets (termed insulitis) of type 1 diabetic donors was not uniformly observed [12]. Analyses of global gene expression profiles of pancreata and islets from the same type 1 diabetic donors revealed that many more pathways were transcriptionally altered in pancreata than in islets, including those for chemotaxis, inflammation, innate immunity, and IFN response [13]. Furthermore, a proteomic analysis of human pancreata found significant upregulation of proteins involved in inflammation from type 1 diabetic donors, as well as autoantibody-positive non-diabetic donors [14]. Together, these data suggest that innate immunity and inflammation of the *exocrine* pancreas may play an important pathogenic role in type 1 diabetes.

Currently, there is no method for identifying pre-diabetic patients at the very early stage of disease initiation, which would be essential for employing potential preventative strategies. If pancreas inflammation can be assessed in the clinic, this may serve as an early indicator of disease initiation. In general, the inflammatory response involves changes in blood vessel and blood flow characteristics. In this study, we chose to use ultrasound imaging technology to visualize and monitor pancreas inflammation, not only because ultrasound is an excellent tool to assess blood vessel and blood flow characteristics, but also because it is a non-invasive tool routinely used in the clinics which will facilitate easier translation of this detection method to the clinics.

We used the virus-inducible BBDR rat model 1) to systematically investigate stage-specific changes in whole pancreas that occur prior to and during autoimmune insulitis by histology, immunoblotting and flow cytometry analyses, and 2) to explore whether ultrasound imaging technology can reveal autoimmune diabetes-specific changes longitudinally. In previous studies, we have carefully defined the stages of autoimmune diabetes pathogenesis in this rat model [15, 16]. The nearly 100% diabetes onset rate upon induction and the highly consistent time course of this model make longitudinal imaging examination possible. To our knowledge this is the first study to visualize pancreas inflammation, *prior to insulitis*, during the progression to autoimmune type 1 diabetes.

## Materials and methods

### Animals

BioBreeding Diabetes Resistant (BBDR) rats were bred at UMASS. Animals were housed in a viral-antibody-free facility in accordance with our Institutional Animal Care and Use Committee. All animal work in this study was approved by the University of Massachusetts IACUC committee (protocol 1766). Anesthesia used was ketamine, euthanasia was by cardiac bleed.

### Diabetes induction

BBDR rats of either sex and 21–24 days old were pre-treated by i.p. injection with polyinosinic:polycytidylic acid (pIC, a viral mimetic) (1–2 μg/g body weight) on three consecutive days (days -3, -2, and -1); pIC (Sigma-Aldrich, St. Louis, MO) was dissolved in Dulbecco’s PBS. Rats received a single i.p. dose of 1x10^7^ PFUs of Kilham rat virus (KRV) on day 0. Control rats received i.p. injections of PBS on the same days. Rats were tested for glycosuria (Clinistix, Bayer, Elkhart, IN) at day 10–11 post-KRV treatment. Diabetes was confirmed by blood glucose >14 mmol/l on two consecutive days (Accu-Chek Aviva, Roche Diagnostics, Indianapolis, IN).

### Pancreatic histology and immunofluorescence staining

Rat pancreata were fixed in 10% buffered formalin and paraffin embedded. Sections were stained with hematoxylin and eosin (H&E) or anti-rat CD8a (BioLegend, San Diego, CA) and guinea pig anti-insulin (Dako, Carpinteria, CA). Briefly, the fixed sections were blocked with PBS-AT (2% BSA grade J and 0.5% Triton X-100 in PBS). Sections were incubated with primary antibody at 4°C overnight. After three washes with PBS, the sections were incubated with secondary antibody at room temperature for 1 hour followed by three washes in PBS. Mounting medium, Vectashield with DAPI, (Vector Laboratories, Inc., Burlingame, CA, USA) was added to the sections. Alexa Fluor 488 and 594 secondary antibodies were from Invitrogen (Carlsbad, CA); isotype controls were from BD Bioscience (San Jose, CA). Images were acquired with a Nikon Eclipse Ti series microscope and analyzed with Nikon Elements image analysis software.

### Isolation of islets and pancreas-infiltrated immune cells

Pancreatic islets from rats were isolated by collagenase digestion as described [17, 18], snap-frozen in liquid nitrogen, and stored at -80°C until use. To recover pancreatic immune cells, all supernatants from the islet isolation procedure were collected and pooled together on ice until centrifuged at 1000 RPM for 15 sec to remove residual exocrine tissue. The clear supernatant was centrifuged at 1500 RPM for 5 min, and the resulting cell pellet containing the pancreas immune cells was washed twice with PBS+0.5% BSA and resuspended in 300ul of MACS staining buffer (PBS-BSA-EDTA).

### FACS analysis of pancreas-infiltrated immune cells

Antibodies for FACS staining included anti-rat CD45, clone OX-1; anti-rat CD3, clone G4.18; anti-rat CD161a, clone 10/78; and anti-rat CD8, clone OX-8 (BioLegend). Intracellular staining was done following the standard protocol, and samples were analyzed on LSRII or FACSCalibur instruments (BD Biosciences). Data analysis was performed with FlowJo (Tree Star, Inc., Ashland, OR) software.

### Immunoblot analysis of isolated islets and whole pancreata

Whole pancreas or isolated islets were recovered from individual rats and lysed with T-PER tissue protein extraction reagent (Thermo-Scientific, Rockford, IL); protein concentrations were determined by bicinchoninic acid protein assay (Sigma-Aldrich). Protein samples were mixed with 4X SDS-PAGE loading buffer, and loaded onto 4–20% precast Tris-glycine gradient gels (Invitrogen, Grand Island, NY). Densitometric analyses were performed with Adobe Photoshop, normalized to actin levels and presented as average±STDEV. Antibodies included rabbit anti-human caspase-3 (Cell Signaling, Beverly, MA); mouse anti-actin (Chemicon International, Billerica, MA) was used as a loading control. Secondary IgG-HRP conjugates were from Santa Cruz Biotechnology (Santa Cruz, CA).

### Ultrasound imaging on large blood vessels and organs of live rats

Vevo 2100 micro-ultrasound system from VisualSonics (Toronto, Ontario, Canada) was used for visualization of anatomical structures down to 30 microns. Rats were individually positioned on a Vevo Imaging Station; isoflurane-based anesthesia delivery, body temperature, electrocardiogram, respiration, blood pressure, and heart rate were monitored throughout the imaging session. Two-Dimensional Ultrasound and Color Doppler were used to identify major arterial and venous structures that feed the various organs, such as aorta, renal artery, superior mesenteric artery, celiac artery, splenic artery, and iliac arteries, as well as the organs including pancreas, spleen and kidney. The 2D imaging was then mathematically rendered into 3D imaging using the software developed by VisualSonics.

### Perfusion study on small vessels/capillaries using ultrasound

MicroMarker Contrast Imaging Mode combined with Non-Linear Contrast Mode was used to quantify small vessels/capillaries of selected organs, including pancreas, spleen and kidney. 50 μL of 1x10^8^ micro-bubbles was perfused over a 3 second time period by intravenous injection into the tail vein using a size A 27-gauge butterfly cannula (Terumo Medical Corporation, Somerset, NJ). The contrast agent (kindly provided by VisualSonics) was lipid-shelled micro-bubbles, 2–3 microns in size, which extends detectability down to 2–3 microns. Perfusion kinetics were assessed by measuring wash-in area, wash-in rate, and peak enhancement.

### Quantification of blood flow in large blood vessel using ultrasound

Blood flow in the blood vessel of interest was quantified by measuring peak systolic and end diastolic velocities using Pulsed Wave Doppler Mode. Resistivity index reflects the vessel resistance to blood flow and was calculated as follows: Resistivity index = (Peak Systolic Velocity—End Diastolic Velocity) / Peak Systolic Velocity.

### Statistical analysis

Statistical analysis was performed by Kaplan-Meier or unpaired t-test using Prism 5 (GraphPad Software, La Jolla, CA); *p* value <0.05 was considered statistically significant.

## Results

### Pancreas inflammation is observed prior to insulitis in diabetes-induced rats

**Fig 1:**
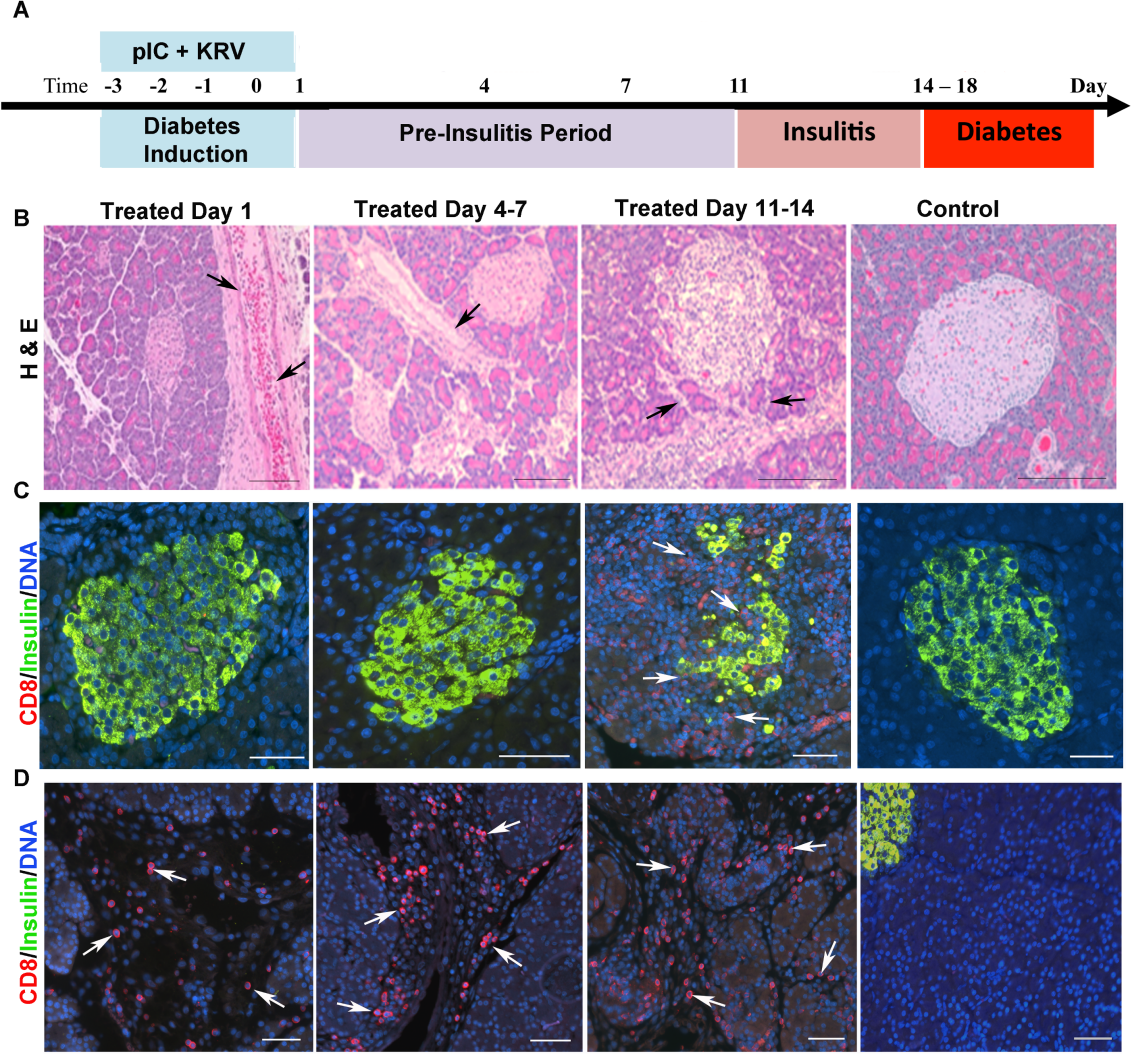
Pancreas inflammation in diabetes-induced rats occurs prior to insulitis while islet morphology is normal. (A) Pancreas tissue was collected from pIC+KRV treated and control rats at the time points indicated and processed for histology and immunostaining. (B) Representative images of H&E stained pancreas sections from pIC+KRV treated and control rats. n = 6 rats/time point; scale bars, 100 um. Arrows indicate vessel/duct dilation. Representative immunofluorescent images of islets (C) and exocrine tissue (D) from pancreas sections of pIC+KRV treated and control rats; CD8 (red), insulin (green), DNA (blue). n = 4 rats/time point; scale bars, 50 um. Arrows indicate CD8+ cells.

We have previously shown that nearly 100% of pIC+KRV treated rats develop diabetes following a highly reproducible time course, with detectable insulitis beginning around 11 days and acute hyperglycemia after 14 to 18 days post-induction [15, 16]. To investigate changes within the entire pancreas, pancreata from diabetes-induced and control rats were collected at selected time points following treatment (Fig 1A). At Day 1, blood vessels and pancreatic ducts within the exocrine tissue of diabetes-induced rats were grossly dilated as shown by H&E staining (Fig 1B). This dilation was still somewhat visible at Day 4–7, although greatly reduced at later time points (Day 11–14).

### CD8+ cells and CD8+CD161+ cells infiltrate the pancreas during the pre-insulitis period

Despite the high heterogeneity of human type 1 diabetes pancreas morphology, a consistent finding is the identification of CD8 T cells as a major constituent when insulitis is present [12]. A further study from these same investigators revealed enhanced CD8 T cell infiltration within the exocrine pancreas of type 1 diabetic individuals as well [10]. To determine whether the pancreata of diabetes-induced rats were also infiltrated by CD8 T cells, pancreas sections from different time points were stained with CD8 antibody. As expected, the majority of islets at Day 11–14, the insulitis stage, had abundant CD8+ cells, and no CD8+ cells were detected in the islets prior to this stage (Fig 1C). In contrast, during the pre-insulitis period, starting at Day 1, the pancreatic exocrine tissue of diabetes-induced rats had a dramatic increase in the number of CD8+ cells compared to the control rats (Fig 1D). Because initially the CD8+ cells were not near the islet areas, the islet (Fig 1C) and CD8+ cells (Fig 1D) are shown in separate views.

**Fig 2:**
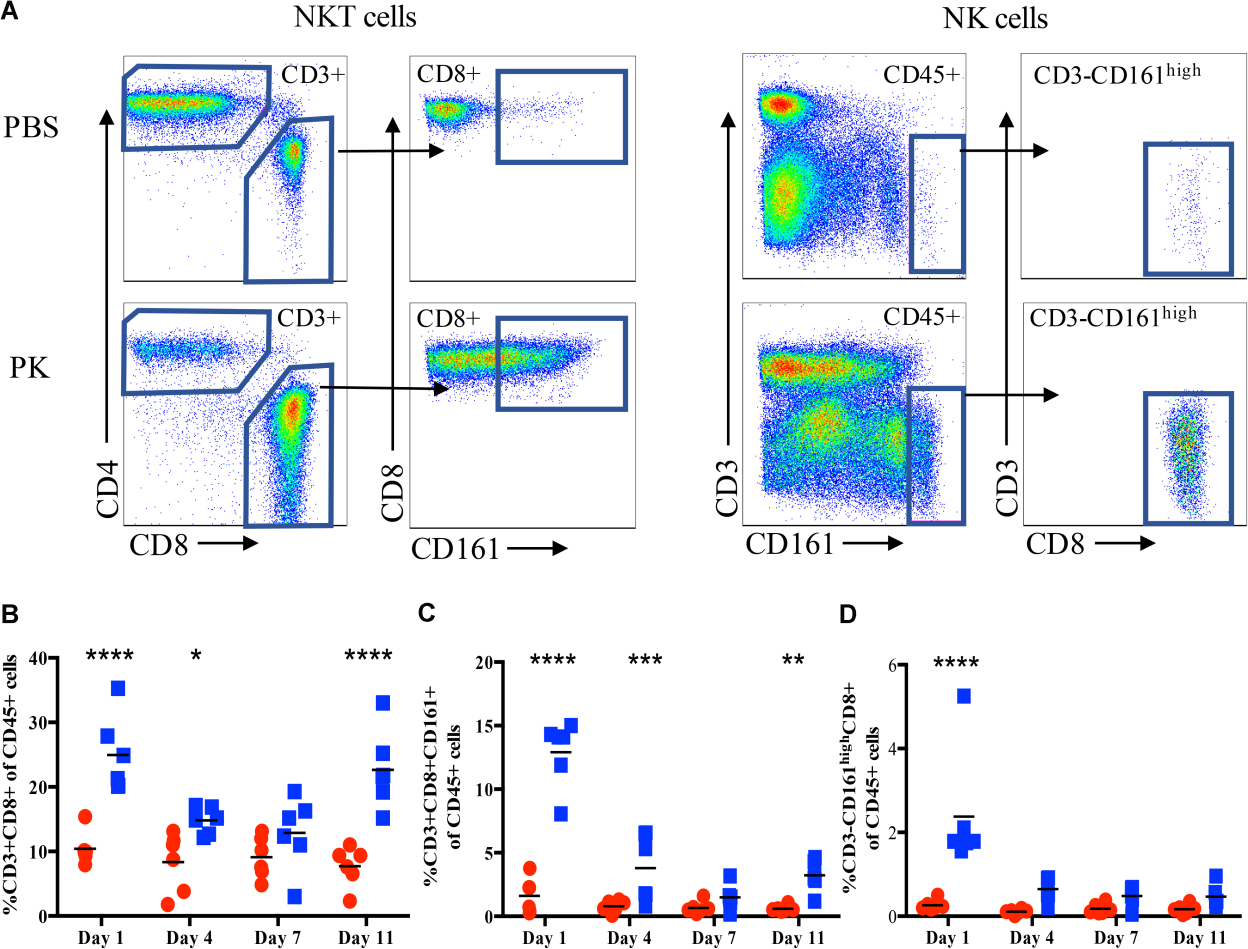
CD8+ cells infiltrate the pancreas at a very early time point after diabetes induction. Pancreas infiltrating immune cells were analyzed at the indicated times from control rats (red circles) and pIC+KRV treated rats (blue squares) by flow cytometry. (A) Representative flow gating for pancreatic supernatant on Day 1. Single, live cells were gated based on expression of CD45; CD45+ cells were further gated on CD3 expression. CD3+ cells were divided into CD4+ and CD8+ cells and expression of CD161 was further analyzed on CD3+CD8+ cells. Additionally, CD45+ cells that were CD3-CD161^high^ were further analyzed for expression of CD8. Shown are percentages of CD8+CD3+ cells of the CD45+ population (B), CD3+CD8+CD161+ NKT cells (C), and CD3-CD161^high^CD8+ NK cells (D): n = 6, each dot represents data from a single rat. Comparison is between control and pIC+KRV treated rats at each time point; *p<0.05, **p<0.01 and ***p<0.001.

To further characterize and quantify the CD8+ cells that were infiltrating the pancreas, the recovered cells isolated from the exocrine pancreas were stained for flow cytometric analysis. Because CD8 is also expressed on NK cells in the rat [19], we included an antibody for CD161 (NK cell marker) as well as CD3 (T cell marker). Representative gating for CD3+CD8+CD161+ NKT cells and CD3-CD8+CD161^high^ NK cells is shown in Fig 2A. Consistent with our immunohistology data, many pancreas-infiltrating immune cells were indeed CD8+. At Day 1 post-induction, compared to control rats the diabetes-induced rats showed a significant increase in percentage of CD8+ T cells (Fig 2B) and NKT cells (Fig 2C). Interestingly, the percentage of both CD8+ T cells and NKT cells returned to control levels by Day 7, but were again significantly increased compared to control rats at Day 11 post-induction, coinciding with the start of insulitis. The percentage of CD3-CD161^high^ CD8+ NK cells in the exocrine pancreata of the diabetes-induced rats were significantly increased only at Day 1 and returned to control levels by Day 4 (Fig 2D).

### Apoptosis in whole pancreata of diabetes-induced rats occurs prior to its detection in islets

**Fig 3:**
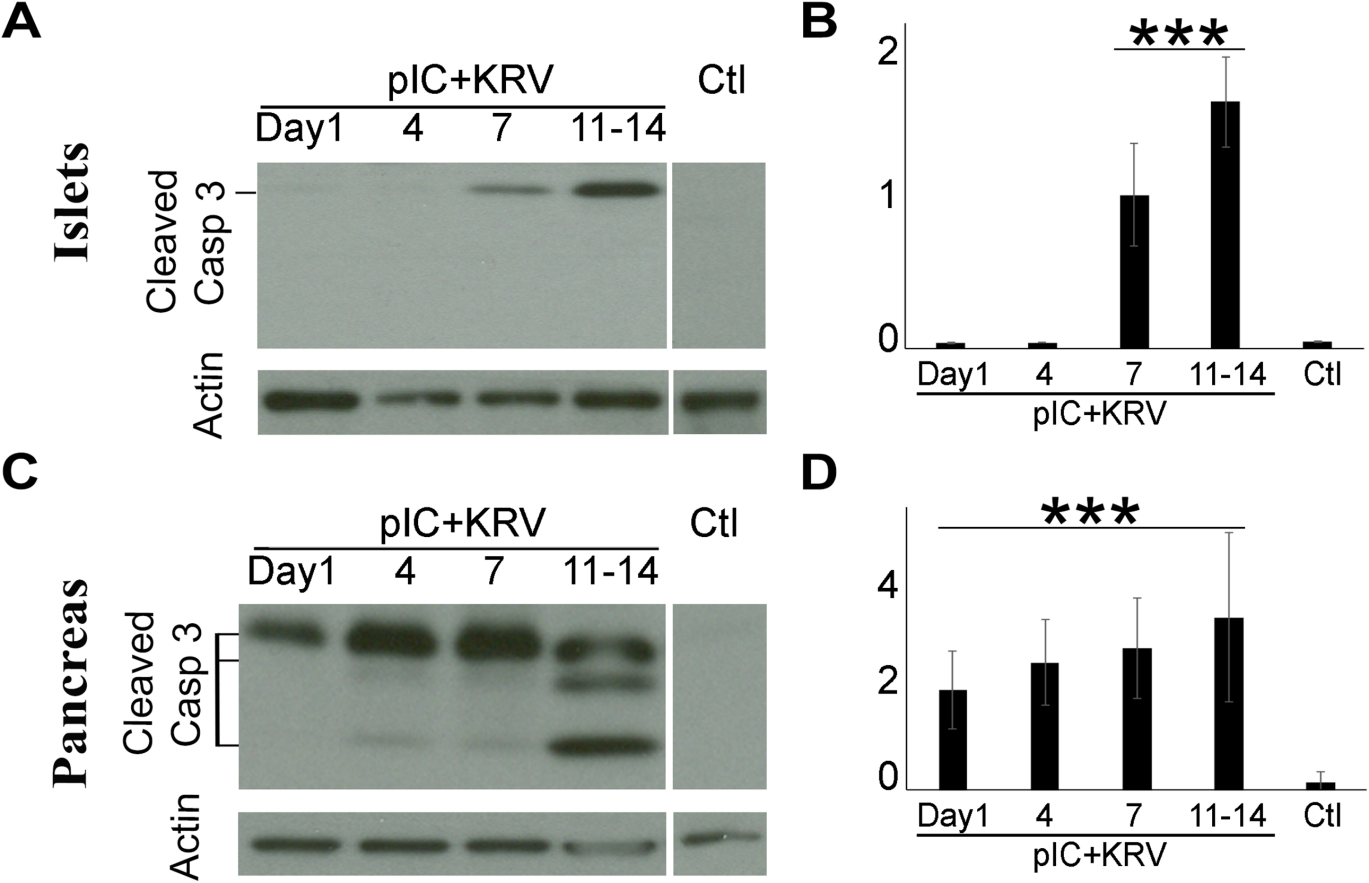
Caspase 3 is activated much earlier in whole pancreas than islets in diabetes-induced rats. Protein lysates from whole pancreata or isolated islets from pIC+KRV treated and control rats were collected at the time points indicated. Representative Caspase 3 immunoblots of isolated pancreatic islets (A) and whole pancreas (C) from pIC+KRV treated and control rats; actin was used as a loading control. Densitometric analyses of the immunoblots from isolated pancreatic islets (B) and whole pancreata (D), normalized to actin. n = 3, error bars show SE. ***p<0.001 for pIC+KRV treated versus control rats.

We have previously shown that islets isolated from diabetes-induced rats undergo ER stress and that the apoptosis marker, active (cleaved) caspase-3, is upregulated prior to insulitis [20]. Consistent with our previous report, active caspase-3 in isolated islets of diabetes-induced rats was first observed at Day 7, with increased apoptosis observed at Days 11–14 when insulitis was evident (Fig 3A and 3B). Here we extend that observation and additionally compare the expression of active caspase-3 in protein lysates from whole pancreas of diabetes-induced rats. Immunoblot analyses of pancreata from diabetes-induced rats showed significant evidence of apoptosis as early as Day 1 post-induction (Fig 3C and 3D). Levels of active caspase-3 from the pancreata of diabetes-induced rats continued to increase at later time points (Days 4 and 7) in the pre-insulitic period, as well as during insulitis at Days 11–14 post-induction. As expected, active caspase-3 was undetectable in isolated islets (Fig 3A) and whole pancreata (Fig 3B) of control rats. These data suggest that the pancreatic exocrine tissues undergo apoptosis well before the islets of the diabetes-induced rats.

### Ultrasound visualization of major blood vessels and the respective organs they perfuse

**Fig 4:**
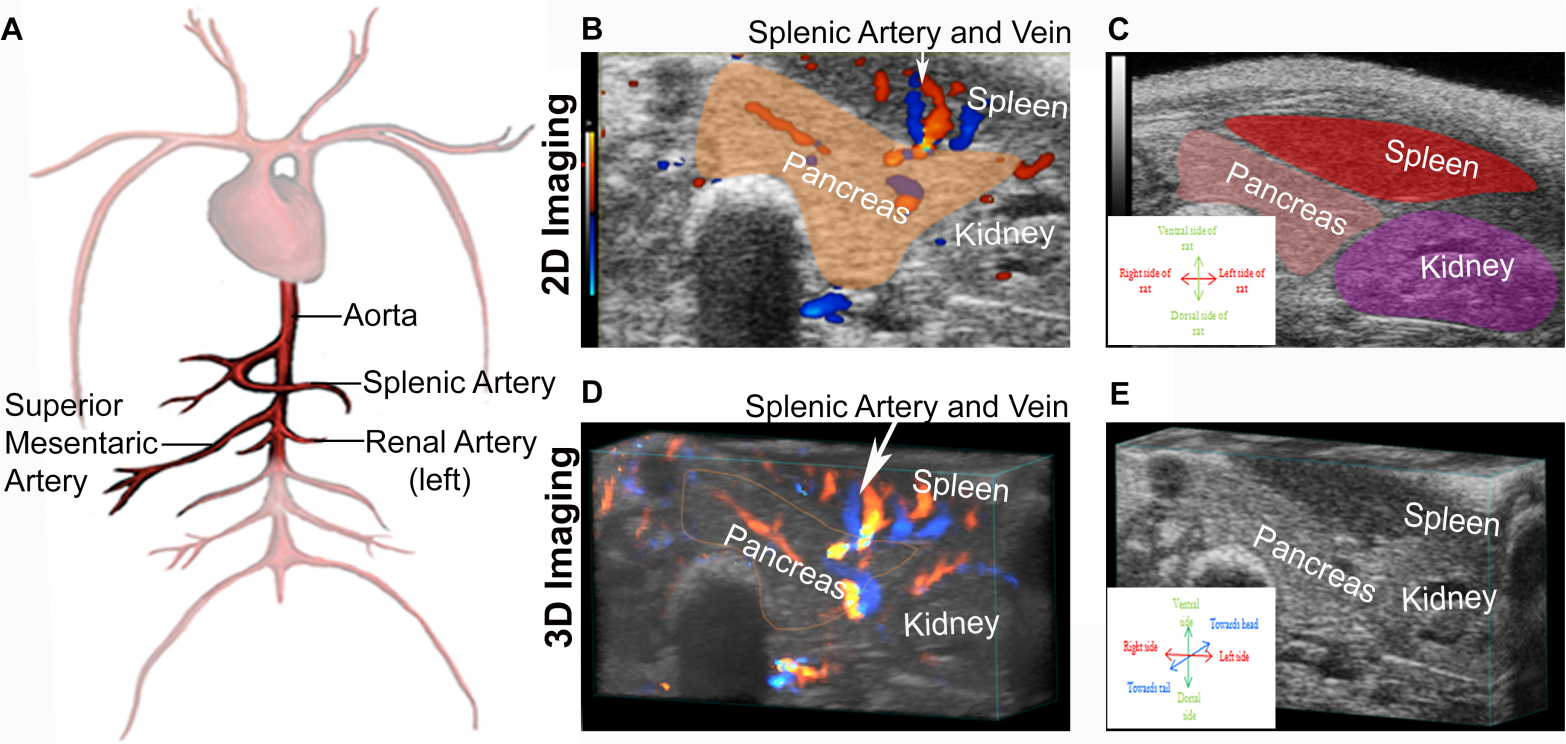
Ultrasound imaging technology can identify various blood vessels and organs, including the pancreas. (A) Schematic drawing of major blood vessels that were used as landmarks to locate the pancreas with ultrasound imaging technology. (B-D) Visualization by ultrasound imaging technology of the pancreas, spleen and left kidney of a representative control rat using the splenic and left renal arteries as location references. Representative image using 2D imaging mode (B-C) and using 3D imaging mode (D-E).

Our H&E histology (Fig 1B) showed pronounced dilation of the blood vessels/ducts in the pancreata of the diabetes-induced rats. To determine if ultrasound technology could provide a useful means to visualize this pancreatic inflammation in a live animal, we first located the relevant major blood vessels and the respective organs they supply/perfuse. Due to its size, the abdominal aorta was the easiest vessel to visualize. As shown schematically in Fig 4A, the splenic, renal, and continuing distally, the superior mesenteric arteries, were then identified. Following the splenic and left renal arteries, the spleen and left kidney were located and used as anatomical landmarks to locate the pancreas by 2D imaging (Fig 4B and 4C). The 3D imaging (Fig 4D and 4E) was then mathematically rendered from the 2D imaging.

### Increased perfusion of pancreatic micro-vasculature in diabetes-induced rats one day post-induction

**Fig 5:**
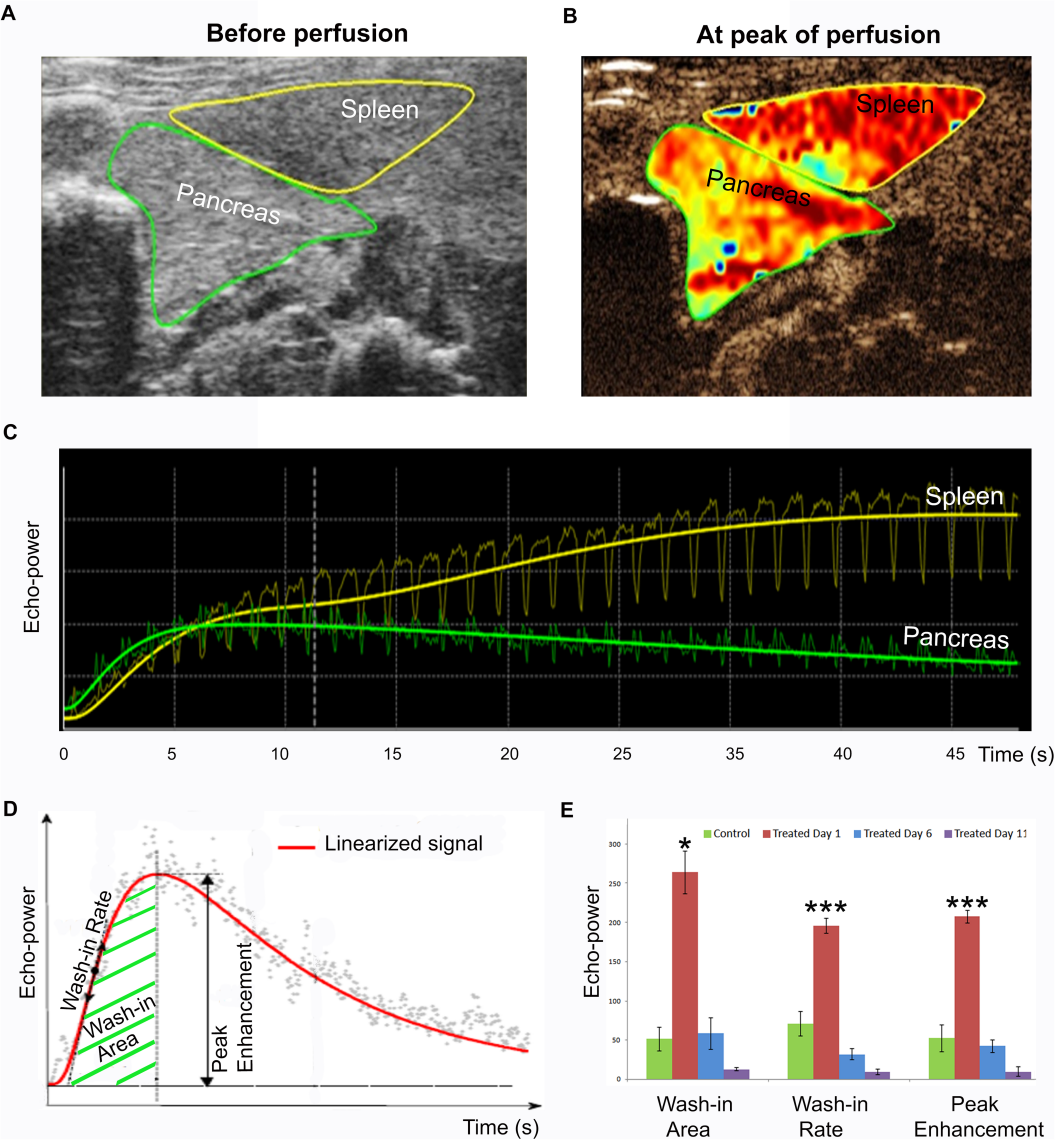
Perfusion of pancreatic micro-vasculature is highly elevated at the earliest stage following diabetes induction. MicroMarker Contrast Imaging Mode combined with Non Linear Contrast were used to generate the ultrasound images shown. Representative ultrasound images from a treated rat are shown (A) prior to micro-bubble injection and (B) at peak of perfusion following injection. (C) Raw perfusion data for the pancreas (green) and spleen (yellow) of the same rat. (D) Schematic diagram of linearized perfusion data indicating wash-in rate, wash-in area, and peak enhancement. (E) Measured wash-in area, wash-in rate, and peak enhancement of pIC+KRV treated and control rats at selected time points; Day 1 (red), Day 6 (blue) and Day 11 (purple) post-induction and age-matched control rats (green). n = 3, error bars represent SE; *p<0.05 and ***p<0.001 for Day 1 pIC+KRV treated rats versus all other groups.

To assess small blood vessel/capillary characteristics within different organs, a combination of ultrasound and perfusion techniques was used. In this study, MicroMarker Contrast Imaging Mode combined with Non Linear Contrast Mode ultrasound technology were used to quantify small vessels/capillaries of selected organs, particularly the pancreas. Rats from both diabetes-induced and control groups were perfused with micro-bubbles by i.v. injection through the tail vein. Representative ultrasound images for a diabetes-induced rat are shown prior to micro-bubble injection (Fig 5A) and at peak of perfusion following injection (Fig 5B). The raw perfusion data for pancreas and spleen from this same rat are shown in Fig 5C; such data are linearized (as depicted in Fig 5D) to obtain the respective wash-in area, wash-in rate and peak enhancement data. The wash-in rate reflects how quickly the micro-vasculature of the area of interest takes to reach peak perfusion, whereas the wash-in area and peak enhancement indicate the relative blood volume in the area of interest. Diabetes-induced rats monitored at Day 1 post-induction had significantly larger wash-in area, higher peak enhancement, and faster wash-in rate than the control rats (Fig 5E). These analyses indicate that the micro-vasculature in the pancreata of diabetes-induced rats was dramatically dilated very early following induction. However, this micro-vascular dilation in the diabetes-induced rats was only observed at Day 1, not at Days 6 and 11 post-induction. Consistent with this, our H&E staining (Fig 1B) also showed the greatest pancreatic blood vessel dilation at Day 1. A representative video shows micro-bubble perfusion through the pancreas of a treated rat (S1 Fig).

### Reduction in vessel resistance of the superior mesenteric artery of diabetes-induced rats prior to and at onset of insulitis

**Fig 6:**
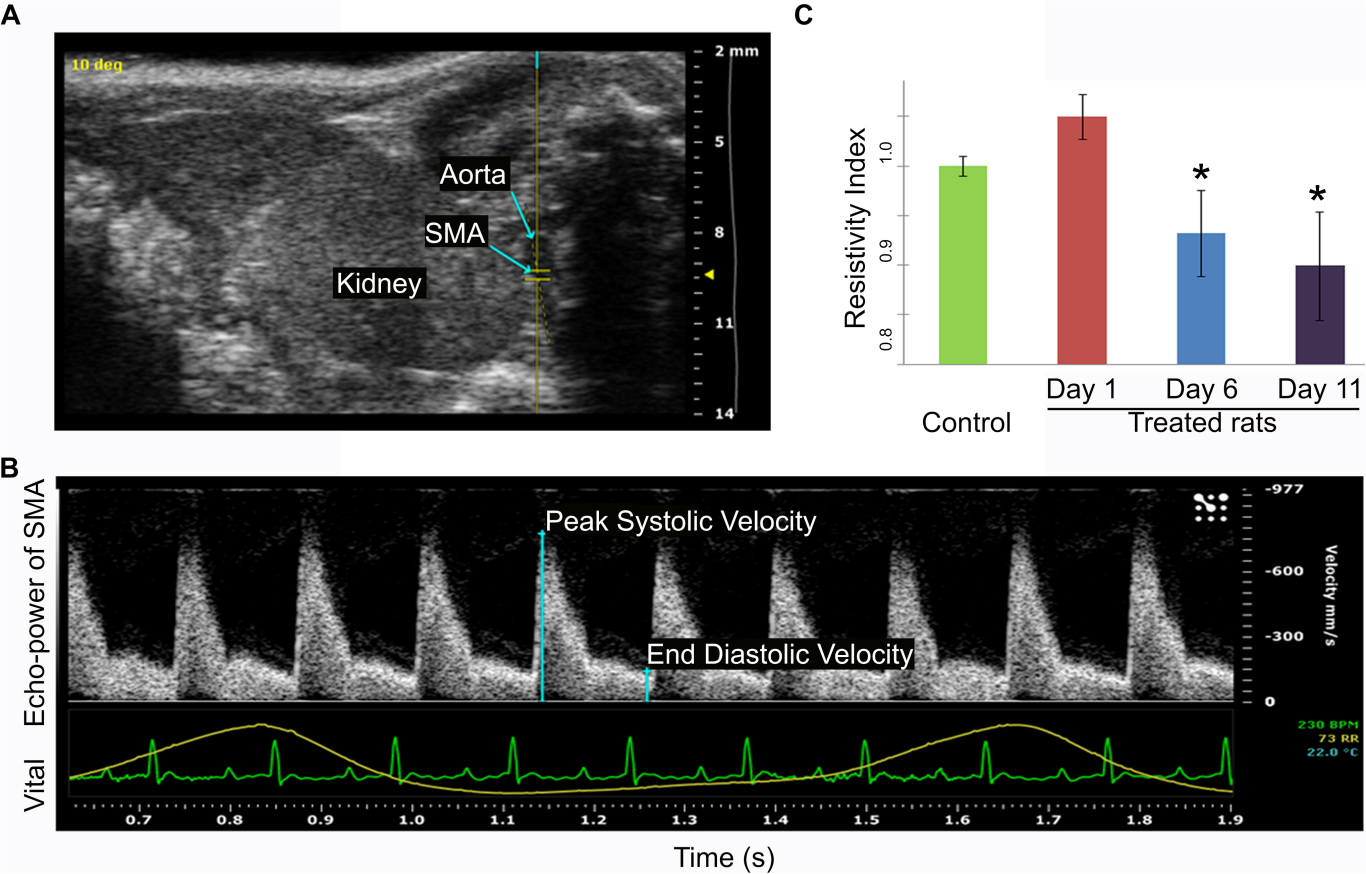
Vessel resistance in the superior mesenteric artery is significantly reduced at later stages following diabetes induction. Ultrasound imaging was first used to locate the superior mesenteric artery (SMA) and Pulsed Wave Doppler mode was then used to measure its peak systolic and end diastolic velocity. (A) Representative ultrasound image showing the location of the SMA, aorta, and kidney. (B) Representative ultrasound image using Pulsed Wave Doppler mode to measure peak systolic and end diastolic velocity of the SMA for calculation of the Resistivity Index. The lower green and yellow lines show vital signs for heart rate and breathing cycle, respectively, from a control rat. (C) The calculated Resistivity Index of the SMA of pIC+KRV treated and control rats at the times indicated. n = 3, error bars show SE; *p<0.05 for Day 6 and Day 11 pIC+KRV treated rats versus Day 1 pIC+KRV treated rats and age-matched control rats.

Although micro-bubble perfusion allowed detection of micro-vasculature changes in the pancreata of diabetes-induced rats, the ideal use of ultrasound as a clinical diagnostic tool would be totally non-invasive, with no injection at all. Ultrasound is a superior method to analyse large blood vessels, therefore to determine whether this technology is able to detect measurable phenotypic changes in large blood vessels prior to diabetes onset, we examined vessel resistance of the superior mesenteric artery of diabetes-induced and control rats. The superior mesenteric artery (SMA), which supplies oxygenated blood to the head and body of the pancreas and the intestine, was identified (Fig 6A). Using Pulsed Wave Doppler mode the peak systolic and end diastolic velocities were measured (Fig 6B) and used to calculate the Resistivity Index, which reflects the blood vessel resistance to blood flow. Using the same rats from the perfusion study above, the Resistivity Index of the superior mesenteric artery in the diabetes-induced rats was found to be significantly lower at Day 6 and Day 11 post-induction compared to the control rats (Fig 6C), indicating that the superior mesenteric artery was inflamed during this period. Importantly, because this decreased Resistivity Index occurs well prior to insulitis (Day 6) and is sustained through the onset of insulitis (Day 11), this may reflect more persistent vascular changes that impact the pancreas *prior* to autoimmunity and diabetes onset.

## Discussion

This study demonstrates that the inducible BBDR rat model is an excellent model to study type 1 diabetes because it recapitulates many pathological characteristics observed in human specimens. While islet morphology appeared to be normal prior to autoimmunity in the diabetes-induced rats, the exocrine pancreas underwent dramatic changes at a very early stage of disease initiation. These changes include dilation of blood vessels and ducts, and the infiltration of CD8+ cells, including NK and NKT cells. Enhanced CD8 T cell infiltration has been reported within the exocrine pancreas of cadaveric type 1 diabetic pancreas specimens [10]; and, in 3 of 6 type 1 diabetic organ donors, the pancreas tissue showed evidence of Coxsackie B4 enterovirus and islet infiltration that was mediated mainly by NK cells [21]. Our study shows increased CD8+ cells within the exocrine pancreas of the diabetes-induced rats well prior to insulitis, suggesting a role for pancreatic inflammation in autoimmune diabetes initiation and progression. In a previous study, we reported that islets undergo cell death via ER stress prior to insulitis, here we show that exocrine pancreas cell death occurred even before islet cell death was detected [20]. Ultrasound imaging showed that exocrine pancreas micro-vasculature was significantly dilated shortly after diabetes induction, and large blood vessel inflammation occurred several days later and was maintained through insulitis. Together, these data demonstrate significant pancreas and blood vessel changes in the diabetes-induced rat prior to autoimmunity—that can be visualized longitudinally by ultrasound imaging technology in living animals.

In a study with the NOD (non-obese diabetes) mouse, MRI imaging with magneto-fluorescent nanoparticles was used to visualize microvascular leakage, an indicator of inflammation at the stage of autoimmunity [22]. Visualization of pancreas inflammation using MRI imaging has recently been attempted in humans using the magnetic nanoparticle ferumoxytol [23]. These investigators found an increase in whole pancreas nanoparticle accumulation, which reflects uptake by macrophages in the inflamed pancreas of recent-onset type 1 diabetic patients. However, ferumoxytol is not approved for patients under 18 years of age, and type 1 diabetes is typically diagnosed in young children. We chose to use ultrasound imaging technology, as it might be a more clinically favorable imaging tool to use with children, and is more cost efficient and convenient than MRI imaging. The micro-bubble we used consisted of a lipid shell with air inside that burst in a few minutes and left no trace afterwards. Currently, this micro-bubble technique is available in clinics in Europe, and is allowed for some clinical applications in the United States. This technique may enable investigation of pre-diabetic stages early in the course of the disease, thus allowing time for potential therapeutic intervention at even earlier stages before disease onset.

Indeed, we show here that ultrasound imaging technology was able to visualize pancreatic micro-vasculature inflammation in the diabetes-induced rats one day following pIC+KRV treatment. In support of this, we previously found that pro-inflammatory IL-1β levels in sera of diabetes-induced rats were significantly increased at the same time point [24]. In humans, multiple pro-inflammatory cytokines, including IL-1β, were significantly higher in children positive for islet autoantibodies [25]. The involvement of the innate immune response and IL-1 regulated genes was also supported by a functional genomics study of human sera [26]. Furthermore, gene expression and genetic association studies support a link between the innate immune response and susceptibility to human type 1 diabetes [27]. Consistent with this, C4d, a marker of complement activation, was also increased in the pancreata of type 1 diabetic donors, and was localized to the endothelium and extracellular matrix surrounding pancreatic blood vessels and ductal structures, as well as the endothelium of pancreatic capillaries [28]. Taken together these studies indicate the presence of pancreatic inflammation during type 1 diabetes.

Besides dilation of micro-vasculature, the inflammatory process can also involve increasing blood flow and vascular permeability. In diabetes-induced rats, large blood vessels (superior mesenteric artery in our study) also had lower resistance prior to insulitis, which shows that blood vessel dilation indicative of inflammation also occurred in larger blood vessels. It is important to note that dilation of large vessels supplying the pancreas of diabetes-induced rats was first detectable by ultrasound at a later time point (Day 6), after the acute micro-vasculature dilation observed at Day 1 had subsided, but still prior to the onset of insulitis. Indeed, Pulsed Wave Doppler mode ultrasound imaging continued to show statistically significant dilation of the superior mesenteric artery that supplies the pancreas through the onset of insulitis (Day 11), indicating that these inflammatory changes may be sustained, rather than merely a transient response. If so, ultrasound technology may prove useful in the clinic for longitudinal studies to distinguish individuals undergoing a transient inflammatory viral infection that resolves without leading to induction of autoimmunity, from those in the early stages of disease development whose pancreas inflammation would persist. In support of this, evidence of ongoing pancreatic inflammation has been reported in autoantibody-positive non-diabetic donors as well as known diabetic donors [14].

Based on the data reported here, we hypothesize that type 1 diabetes development in some genetically-susceptible individuals may start with an environmental perturbation, such as a viral infection, and this perturbation may induce inflammation both systemically and in multiple organs, including the pancreas. We speculate that pancreas inflammation, in proximity to islet β-cells, may have direct pathological effects on islet health, such as ER stress, and progress to some *initial* β-cell death as we have previously observed [20]. In turn, the signals released from these stressed and dying β-cells, in conjunction with an unresolved innate immune response, may reach a critical ‘threshold’ for autoimmune activation in those individuals genetically at-risk, and lead to insulitis and a further substantial autoimmune-mediated destruction of β-cells. In this scenario, the individual’s innate [24] immune response to the virus may be more important in the initial β-cell destruction than a direct role for the virus itself.

In support of this, in our previous studies, we compared the serum levels of inflammatory proteins in rats treated with pIC+KRV or pIC+H1 virus [24, 29]. Although H1 and KRV are both parvoviruses with 98% sequence identity, pIC+H1 treatment does not induce diabetes [30]. Briefly, on day 1, both pIC+KRV and pIC+H1 treated rats had a ~15-fold increase in serum haptoglobin, an acute phase protein. Although haptoglobin levels in pIC+H1 treated group subsequently declined, the levels in pIC+KRV treated rats remained elevated through day 7. The inflammatory cytokines, IL-1β and IL-6, were increased in rats treated with pIC+KRV, but levels in pIC+H1 treated rats were not significantly different from those of PBS treated controls. Also, serum levels of IL-12 in rats with pIC+KRV treatment were elevated and remained elevated at later time points, whereas pIC+H1 treatments failed to induce IL-12 at any time point. Furthermore, pancreas histology of pIC+H1 treated rats did not show the typical dilation of blood vessels and ducts, and CD8 staining was similar to the PBS treated control group. In summary, the data suggested that pIC+KRV treated rats had greater innate immune activation, and this activation persisted for a longer period than in the pIC+H1 treated group.

Indeed, the role of innate immunity in the *development* of type 1 diabetes is supported by genetic and functional studies in both animal models and humans [25–27, 31–36]. Importantly, increased innate immune activity has been reported in genetically at-risk children even *prior to* detection of serum autoantibodies [37, 38]. In view of this, we envision that the most effective use of ultrasound to identify at-risk children at the very earliest stages of disease development would involve assessment of pancreas inflammation when these children present with a fever, such as may occur with a presumed viral infection. These at-risk children would be monitored by ultrasound again a few weeks after the fever has resolved to identify persistent pancreas inflammation. If pancreatic inflammation continues, these children could be more closely examined for other indicators of impending type 1 diabetes, such as the presence of serum autoantibodies. Ideally, this would also be an appropriate time to begin preventative treatments, when they become available.

## Conclusions

In conclusion, ultrasound is an excellent non-invasive tool to visualize changes in blood vessel and blood flow that are characteristic of an inflammatory response. Our data show that sustained pancreas inflammation prior to disease onset can be detected by ultrasound in our diabetes-induced rats. For research purposes, the ability to longitudinally monitor pancreas changes in animal models of type 1 diabetes is critical to interrogating the pancreas’ role throughout disease progression, as well as to identify potential treatments that facilitate pancreas health. In combination with perfusion techniques, ultrasound also allows assessment of small blood vessels/capillaries in the pancreas during type 1 diabetes pathogenesis. Currently, this contrast ultrasound technique (e.g., with micro-bubble injection) is available in clinics in Europe, and is cleared by the Food and Drug Administration for some clinical applications in the U.S. Importantly, due to its established use as a safe and effective clinical diagnostic tool, ultrasound technology could be explored as one methodology to aid in the early prediction of type 1 diabetes in the at-risk population, and to assess early treatment strategies that may be developed to prevent type 1 diabetes.

## Supporting information

S1 Fig Representative video showing microbubble perfusion through the pancreas of a diabetes-induced rat. MicroMarker Contrast Imaging Mode combined with Non Linear Contrast mode were used to generate the ultrasound images shown. Video shows a time lapse of 426 seconds following microbubble injection.
(MP4)
